# Defining success together: a collaborative approach for client and provider-engaged outcomes indicator development in Ontario

**DOI:** 10.1186/s40900-025-00832-x

**Published:** 2026-01-07

**Authors:** Melissa Perri, Katherine Rudzinski, Adrian Guta, Jessica Xavier, Gillian Kolla, Soo Chan Carusone, Carol Strike

**Affiliations:** 1 Dalla Lana School of Public Health, University of Toronto, 155 College Street, Toronto, ON M5T3M7 Canada; 2 MAP Centre for Urban Health Solutions, St. Michael’s Hospital, 209 Victoria St, Toronto, ON M5B 1X3 Canada; 3 School of Social Work, University of Windsor, 167 Ferry Street, Windsor, ON N9A0C5 Canada; 4 Faculty of Medicine, Memorial University of Newfoundland, 39 Clinch Crescent, St. John’s, Newfoundland, NL A1B3V6 Canada; 5 McMaster Collaborative for Health and Aging, McMaster University, 1400 Main St W, Hamilton, ON L8S1C7 Canada

**Keywords:** Outcome indicators, Collaborative research methods, People who use drugs, People living with HIV

## Abstract

**Background:**

While there is growing inclusion of people who use drugs in research and policy forums, they are typically excluded when program outcome indicators are determined. We discuss the methods, results, and lessons learned from two projects that engaged people who use drugs, who are living with or at risk of HIV, and service providers in collaborative outcome indicator development.

**Methods:**

Project 1 was conducted with stakeholders of a future day health program and project 2 with stakeholders of safer opioid supply program. For both projects, we included (1) capacity building about the definition and measurement of outcome indicators with stakeholders; (2) collection of stakeholder perspectives about important outcome indicators; (3) rating/selecting proposed outcome indicators; and (4) analyzing and disseminating findings regarding recommended outcomes. We collected and analysed all suggested outcome indicators using content analysis techniques. As a team, we reflected on the lessons learned from our projects.

**Results:**

For study 1, we recruited *n* = 18 clients who participated in up to 4 sessions, and 10 staff/clinicians and 3 leadership members who participated in one session each. For study 2, 8 program clients, 7 internal program providers, and 8 external agency service providers participated in one session each. During the sessions in both projects, participants were shown the mandated outcome indicators for the respective programs, and each group emphasized the need to measure social outcomes in addition to health outcomes, with clients in both studies preferring qualitative interviews for collecting outcome data .Domains of outcome indicators which were highlighted in study 1 include quality of life; mental health; satisfaction with pain management; access to harm reduction services; social connectedness; and ease of readmission for those with frequent episodic illnesses. For study 2, the highlighted outcome domains included experiences of social isolation and connectedness, experiences of discrimination in health and service setting, changes in self-care and leisure activities, and experiences of grief and loss.

**Conclusions:**

Our experiences demonstrate the value of an interactive, participant-driven approach to outcome indicator development that integrates capacity-building to foster meaningful engagement. Across both projects, clients, staff, and leadership contributed valuable insights that enhanced future program evaluation, with participants generating practical, inclusive indicators—some of which were incorporated into electronic medical records and outcome surveys to better capture client well-being. We also identified important lessons about session structure and virtual engagement, finding that condensed sessions can increase accessibility and diversity of input, while virtual formats offer new opportunities for inclusion, but require additional support to engage structurally vulnerable participants meaningfully.

## Abstract (plain language summary)

People who use drugs are included in research and policy conversations but are rarely involved in deciding how to measure the success of the programs they use. We conducted two projects: one for a future day health program and another for a safer opioid supply program to identify meaningful outcome indicators for each program. In both, we provided training to help participants understand what outcome indicators are and how they are used, collected their ideas on what outcomes matter most, supported them in rating those ideas, and analyzed and shared the findings. In Project 1, 18 clients and 10 staff and 3 managers participated. In Project 2, 8 clients, 7 internal staff, and 8 external service providers participated. In both projects, participants emphasized the importance of measuring social outcomes—like connection, self-care, and well-being—in addition to health outcomes. These projects show the value of a participant-driven approach to outcome indicator development that includes training and reflection. Participants generated practical and inclusive indicators, some of which were adopted in program surveys and electronic records. We also learned that shorter sessions can improve access and that virtual sessions can expand participation, but extra support is needed to include people facing housing or technology barriers.

## Background

In recent decades, engaging stakeholders in the design and delivery of research and evidence-based programming has gained momentum [1–6]. Client engagement throughout the research process has been associated with increased knowledge, empowerment, improved program sustainability and resource mobilization, and more relevant policy development [4, 6–10]. Ensuring client engagement in program outcome indicator development, particularly among groups who have been made vulnerable by societal structures (e.g., people living with HIV, people who use drugs), will contribute to the development of effective programming and monitoring. While there is growing inclusion of people who use drugs in research and policy forums [11–13], they are typically excluded when program outcome indicators are designed [14], despite evidence showing the value of community engagement [9].

The lack of involvement in program outcome indicator development among communities of people who use drugs may contribute to the use of measures that fail to capture the factors influencing client engagement, retention, and success—highlighting the risk that what gets measured may not always reflect what truly counts. In this paper, we use the term *client* to be inclusive of both individuals who access health care services as *patients* and those who engage with social, harm reduction, or community-based programs that may not involve medical treatment. While *patient* typically refers to someone receiving clinical or medical care, *client* encompasses a broader range of service users across health and social support systems.

We discuss our methods, results, and some lessons learned from two community-based research projects that engaged people who use drugs, who are living with or at risk of HIV, and service providers in collaborative outcome indicator development. Both of these projects worked with groups who have been made marginal by their health and/or substance use. We approached these studies using a community-based research (CBR) framework, a collaborative approach in which community members, service organizations, and researchers work as equal partners to address issues identified by the community. CBR values lived experience as expertise, emphasizing shared decision-making, co-learning, and the co-production of knowledge that supports local action and social change [15–17]. Rooted in traditions of participatory action research and increasingly applied in public health and HIV sectors, CBR has taken diverse forms across different settings. Within harm reduction research specifically, CBR has been widely used to engage people who use or inject drugs as knowledge holders/users, recognizing their experiential expertise and the importance of centering their perspectives in program development and policy advocacy [18–20]. Given the centrality of lived experience in harm reduction and HIV work, meaningful engagement of people who use drugs is widely recognized as essential for producing ethically grounded, contextually relevant, and actionable evidence. In this tradition, our collaborative outcome indicator development approaches in both projects operationalized CBR principles by involving clients and service providers in identifying and refining outcome measures that reflected their priorities, realities, and definitions of meaningful change. Each project aimed to identify and recommend outcome measures valued by both clients and service providers, ensuring programs could incorporate these meaningful metrics into their evaluation frameworks.

### An overview of collaborative indicator development models

Defining and measuring indicators that relate to specific program outcomes is critical for determining whether a program’s objectives have been met, understanding the well-being of a community, and identifying key areas for improvement [21]. However, health outcome indicators continue to be predominantly defined by external institutions and researchers [22]. This approach can contribute to the measurement of outcomes that do not reflect what a community hopes a program will achieve. The practice of developing ‘indicators’ collaboratively has been used in a variety of fields, and has been described to improve program engagement, community inclusion, and program sustainability [23–25].

The development of patient-reported outcome measures (PROMs) and patient-reported experience measures (PREMs) offers two important approaches to generating indicators that reflect the perspectives of service users. PROMs provide standardized information on a patient’s self-assessed health status, functioning, or quality of life, while PREMs capture patients’ experiences of care, including communication, respect, and service delivery processes [26, 27].Within health and social care, PROMs and PREMs have been positioned as tools that support patient agency, strengthen communication between patients and providers, and improve the responsiveness and effectiveness of services [26, 28, 29]. Increasingly, scholars have argued that these measures can help illuminate dimensions of care that might otherwise remain invisible within traditional clinical or administrative data systems [30, 31].

Within substance use services, however, the PROM/PREM literature remains comparatively underdeveloped, with several notable gaps. Although recent work highlights the potential for PROMs and PREMs to enhance dialogue between providers and people who use drugs and to identify outcomes that matter to these populations [30–32], the development of these measures has been concentrated largely within treatment settings rather than harm reduction contexts. Moreover, the level of service-user involvement in indicator development has often been limited, typically occurring through consultation on pre-designed items rather than through sustained co-creation. Much of the existing research is also quantitative and standardized in orientation, emphasizing psychometric testing over experiential or participatory dimensions [33]. A recent scoping review of PROMs/PREMs in substance use disorder treatment services illustrates this tendency, noting that most available measures focus on measurement validation and outcome monitoring rather than collaborative design [33]. Similarly, van der Sterren et al., in their review of PREMs in alcohol and other drug treatment services, identified 30 measures, yet found that people who use drug involvement in measure development was generally limited, often restricted to one-off interviews or advisory input rather than sustained collaborative design [34]. Only 4/30 measures identified were developed specifically for harm-reduction settings (e.g., needle and syringe programs, drop-in services, or supervised consumption sites) [34]. Efforts to define indicators for harm reduction programming specifically have emerged [35, 36], but without comprehensive stakeholder involvement to identify outcome and experiential priorities for program engagement, such indicators risk overlooking the complexity and innovation of these programs. Collaborative development is therefore essential to ensure indicators are contextually relevant and reflective of community priorities.

Collaborative outcome indicator development practices align with principles of community-based research [37, 38]. Evidence has demonstrated how, when applied appropriately, these methods of research can empower communities of people who use drugs and work towards building community capacity, while reducing stigma (e.g., stigma surrounding drug use, experiences of homelessness, HIV status) and hierarchies of power [19]. By centering the voices of people who use drugs in the design of outcome indicators, we help ensure agency and relevance in the evaluation process and in the implementation of support services. This approach not only empowers participants by recognizing their expertise, but also actively challenges stigma by positioning their lived experience as a source of knowledge. Following these research methods, collaborative practices invite community members to participate in determining outcome indicators, to ensure that program success is measured with outcomes considered important and practical for those who will use and be most impacted by the program [39–41]. Collaborative outcome indicator development practices have the potential to recognize diverse community needs and values as well as minimize the historical and ongoing exclusion of certain groups from research and program design, delivery, and evaluation [7, 22, 23]. In addition, this participatory approach centers societal and cultural norms in every stage of program delivery (i.e., program conception to implementation, evaluation, and outcome assessment) [42].

Core components of the collaborative outcome indicator development model are (1) capacity building about the definition and measurement of outcome indicators with future/current clients and service providers (hereafter stakeholders) of the program for which indicators are being developed; (2) gathering perspectives of stakeholders about important outcome indicators; (3) facilitating a process of rating/selecting proposed outcome indicators; and (4) analyzing and disseminating findings regarding recommended outcomes [22, 39, 41]. Collaborative indicator development practices incorporate capacity building activities about outcome indicators through educational materials designed for stakeholders [22, 39, 41]. The goal of these activities is to increase relevant stakeholders’ knowledge about the purpose of indicators; the different types of indicators; the different methods for selecting indicators; and the characteristics of an effective indicator [22, 39, 41]. Once stakeholders have developed an understanding of collaborative outcome indicators, their perspectives about what indicators are relevant and useful are gathered [43]. These perspectives can be gathered using varying methods including focus group discussions, one-on-one interviews, or written survey-style responses. Following this, stakeholders rank proposed outcome indicators from lowest to highest relevance and priority [44, 45]. In certain situations, program staff and leadership may also engage in ranking proposed outcome indicators. The research team then uses these ranked orders to provide an overall analysis to program leadership to discuss the dissemination of session findings [44, 45]. This paper presents two projects that piloted different collaborative approaches to indicator development, aimed at identifying meaningful ways to evaluate the success of planned and ongoing services.

## Methods

### Project 1 - Collaborative outcome indicator development for a day health program

The aim of this project was to build the capacity of clients, clinical staff, managers, and senior leaders, to develop a set of community relevant health outcomes and process indicators for a forthcoming day health program. At the time of the study, the hospital had 13 inpatient beds and served approximately 100 clients as outpatients. This new program was approved for implementation at an existing specialized care hospital offering inpatient care and outpatient services for people living with HIV, the majority of whom used drugs (PLHIV-WUD). As a requirement of government funding, the forthcoming day health program was required to report government mandated outcome indicators. However, the senior management team wanted to broaden the focus to capture and monitor community-relevant outcomes of the day health program.

### Overview of project 1 sessions

Project 1 employed a community-based research approach, including a research assistant with lived experience of HIV who participated in all aspects of the project and as one of the session facilitators. For clients (i.e., current or former hospital inpatients), we hosted a set of four sequential group sessions to build capacity for outcome indicators and to solicit their opinions about the types of outcomes to measure for the day health program (see Table 1). Clients were recruited for each session (aimed to have 6 to 10 participants per session) via advertisements posted within the hospital as well as flyers distributed by staff and word of mouth. Clients were invited to attend as many of the four sessions as they were able. Eligibility criteria included: living with HIV and currently admitted or had been admitted as an inpatient at the study hospital within the last year. We designed the sessions using key tools for understanding and mobilizing indicator development (see for example: [46–49]) with each session intentionally building upon the previous ones. Activities included: a presentation about what is an outcome indicator and how are these measured; a presentation of funder mandated outcome indicators for the day health program; brainstorming to explore participants’ desires and needs for the day health program and then identifying and prioritizing (using dotmocracy [50]) outcome and process indicators for the program [51]. We obtained verbal consent at each session. Each participant filled out a socio-demographic questionnaire at the first session they attended. Sessions were held in-person, lasted approximately 1.5–2 h, and were co-facilitated by the research coordinator (KR) and a research assistant with lived experience of HIV. Snacks, beverages, and an honorarium of $30 CAD and two transit tickets were provided at each client session.

**Table 1:** Project 1 - sequence of sessions

Number	Group	Topics	Mode
1	Clients	• Introduction/ice breaker	Interactive discussion
		• Health indicators – a brief overview • Social determinants of health – a brief overview	Didactic
		• Case study – identify health indicators	Break out group discussion
		• How are health indicators uses and by whom	Didactic
2	Clients	• Ice breaker/introductions • Review of previous client session content	Interactive discussion
		• Sources of health data • Overview of pros and cons of methods to collect health	Didactic
		• Preferred methods to collect health data for the day health program	Dotmocracy
3	Clients	• Ice breaker/introductions • Review of previous client session contents, verify accuracy of lists	Interactive discussion
		• Planned health indicators for day health program – overview	Didactic
		• Health domains that are most important for clients • Important things the day health program must do to be successful • Indicators that the program is operating successfully	Brainstorm lists
		• Suggest additional client relevant indicators	Brainstorm list and prioritize using dotmocracy
4	Staff	• Introductions • Health indicators – a brief overview • Planned health indicators for day health program – overview	Interactive/didactic
		• Suggest additional indicators for the day health program	Brainstorm list
		• Review of client suggested indicators	Review, refine brainstorming list and prioritize using dotmocracy
5	Clients	• Ice breaker/introductions • Review of previous client session contents, verify accuracy of lists	Interactive discussion
		• Review client priorities for health indicators • Review service provider priorities for health indicators	Didactic
		• Compare and contrast lists • Identify what is missing from the combined lists	Interactive discussion/consensus
		• Identify key health indicators for new day health program	Dotmocracy
		• Identify who and how often data should be collected	Interactive discussion/consensus
6	Senior management	• Introductions • Health indicators – a brief overview • Planned health indicators for day health program – overview	Interactive/didactic
		• Suggest additional indicators for the day health program	Brainstorming list
		• Review of client- and staff- suggested indicators	Review, refine brainstorming list and prioritize

For staff/clinicians (e.g., nurses, social workers, recreational therapists, case managers) and the senior leadership/manager team, we recruited those who would be working in/with the forthcoming day health program using the hospital listserv. For each group, we hosted one session where the activities, as described above, were condensed into a single, long session. Verbal consent was obtained from staff and managers/senior leadership at the start of the sessions. Snacks and beverages but no honoraria were provided for staff and management sessions.

All sessions were hosted from May through June 2017, audio-recorded, transcribed verbatim, and corrected for accuracy. An overview of the content of each session is provided in Table 1. At the close of each session pictures were taken of the lists and dotmocracy results to help facilitate analysis. Research Ethics Board (REB) approval was obtained for this study from the University of Toronto REB (Protocol #34127) prior to data collection.

### Session analysis

The research team (KR, CS, AG, SCC) reviewed and consolidated all responses provided by each stakeholder group creating separate client and staff/ clinician/ recommended outcome indicator lists. We then combined these two lists with the government mandated indicators, removing any duplicates, to create a “master list” of indicators. To support the development of these lists, we applied a conventional qualitative content analysis [52] to session transcripts and indicator-list photos. The research team manually highlighted key ideas, grouped related concepts into inductive categories using participants’ own language, and collaboratively refined these categories to reflect areas of convergence and divergence across stakeholder perspectives. During the last client consultation session, the client group refined the ‘master list’, added additional indicators they deemed appropriate, and used a dotmocracy exercise, allocating sticky dots to the outcomes and data-collection approaches they viewed as most important, to identify process and outcome measures they felt were priorities. This helped to identify preferred methods (e.g., interview vs. survey), timing, and frequency for collecting data related to these outcomes. For the session with the senior leadership/management team, the research team presented a final version of the ‘master list’ refined by the client group to the senior leadership/management team, who were asked to suggest additional indicators but not to remove indicators suggested by the other groups.

### Project 2 - Collaborative health indicator development for a safer opioid supply program

The aim of the project at the safer supply program was to build the capacity of its stakeholders to recommend health outcome indicators that could add to its existing outcome indicator survey that the program administered every six months. The safer supply program provided prescriptions of pharmaceutical-grade opioids to individuals who were at high risk of experiencing an overdose, and used unregulated opioids (primarily unregulated fentanyl) [53]. While it would have been preferable to include a peer co-facilitator in Project 2, this was not possible due to the constraints of the COVID-19 pandemic at the time. Frequent changes to social distancing guidelines, limited access to reliable internet among potential peer research assistants, and the program’s inability to support remote participation created significant logistical barriers to the training and integration of a peer team member. At the time of this study, the program had enrolled 150 clients.

The outcome indicator sessions were held with clients, internal program service providers (e.g., nurses, counsellors), and external agency service providers (e.g., social workers) of the safer opioid supply program. These sessions were held as part of a larger program evaluation project [54]. For project 2, we facilitated one indicator development session per stakeholder group. We held only one session for a variety of reasons, including: (1) in light of COVID-19 pandemic indoor occupancy restrictions, hosting one session per group ensured we did not reduce capacity for the organization to see clients while we conducted the project; (2) experience from project 1 that suggested we might be able to shorten the sessions and achieve similar results for all stakeholder groups; (3) concerns about being able to re-engage multiple participants who were experiencing varying forms of vulnerability (e.g., housing insecurity); (4) timeliness of input for a management team interested in quickly developing an outcome monitoring system required by their funder; and (5) to reduce the space burden on safer opioid supply program of multiple sessions per group.

### Overview of project 2 sessions

Participants were recruited using flyers which were posted within the safer supply program and through referral from the program leadership team. Those interested were invited to contact the study team by email. The safer supply program staff helped any client who did not have access to email to contact the team. Only current clients and internal/external service providers were eligible to participate. Sessions included an ice breaker, interactive discussion of the above-mentioned content, and an overview of health indicators, overview of different sources of data and data collection methods (see Table 2).

**Table 2:** Project 2 - sequence of sessions

Number	Group	Topics	Mode
1	Clients	• Introduction/ice breaker/consent/ demographic survey	Interactive discussion
		• Perceptions on factors which influence health, outcomes relating to health services, and changes to safer supply program clients because of the program	Break out group discussion
		• Rating of safer supply client changes	Dotmocracy
		• Perception on aspects of the program that can help safer supply clients make desired changes	Break out group discussion
		• Rating of program aspects which are beneficial for clients	Dotmocracy
		• Overview of health indicators	Didactic
		• Perceptions on what indicators will demonstrate safer supply program success	Break out group discussion
		• Overview of different types of sources of data	Didactic
		• Rating of data collection methods	Dotmocracy & interactive discussion
2	Internal Providers	• Introduction/ice breaker/consent/ demographic survey • Review of previous client session content	Interactive discussion
		• Perceptions on factors which influence health	Break out group discussion
		• Overview of definitions of health and health indicators	Didactic
		• Case study 1 – applying health indicators to a social services program	Break out group discussion
		• Perceptions & rating of aspects to measure if the program is ‘working well’ for clients	Dotmocracy
		• Overview of existing indicator survey being implemented in the safer supply program	Didactic
		• Perceptions & rating of aspects to measure if the program is ‘working well’ for service providers and at a systems level	Dotmocracy
		• Overview of different types of sources of data	Didactic
		• Rating of data collection methods	Dotmocracy & interactive discussion
3	External Providers	• Introduction/ice breaker/consent/ demographic survey • Review of previous client session content	Interactive discussion
		• Perceptions on factors which influence health	Break out group discussion & Jamboard
		• Overview of aspects of definitions of health and health indicators	Didactic
		• Case study 1 – applying health indicators to a social services program	Break out group discussion & Jamboard
		• Perceptions & rating of aspects to measure if the program is ‘working well’ for clients	Jamboard & Dotmocracy
		• Overview of existing indicator survey being implemented in the safer supply program	Didactic
		• Perceptions & rating of aspects to measure if the program is ‘working well’ for service providers and at a systems level	Jamboard & Dotmocracy
		• Overview of different types of sources of data	Didactic
		• Rating of data collection methods for clients and service providers	Zoom Survey & interactive discussion

All sessions were held in November of 2021 and co-facilitated by CS and AG, who have decades of experience working with people who use drugs and conducting community-based research projects. Sessions with clients and internal service providers were held in person and for external agency providers were held on Zoom. Participants were given a study information sheet, provided verbal consent before each session, and filled out a socio-demographic form. Each session lasted approximately 1.5 h. Clients received a $30 CAD honorarium for participation and snacks during the session.

Session activities included: a presentation about what is an outcome indicator and how are these measured; a presentation of funder mandated outcome indicators for the safer supply program; brainstorming to explore participants’ desires and needs for the safer supply program and then analyzing these to identify and prioritize (using dotmocracy [50]) outcome and process indicators for measuring and monitoring the program. For sessions which were held in person, participants were asked to brainstorm answers to discussion questions verbally and the answers were recorded on easel paper. Using a dotmocracy approach, participants were given 2 sticky dots and asked to place these next to answers they perceived to be most important. For the session held remotely, an online whiteboard was used to record answers to the questions with participants marking a digital x next to answers they perceived to be most important. Each session was audio-recorded and transcribed. Pictures were also taken of all answers to brainstorming questions to facilitate the analysis process. The University of Toronto REB provided approval for this study (Protocol #46493).

### Session analysis

Using content analysis techniques, the research team (MP, CS, AG) reviewed and consolidated all responses provided by each stakeholder group. We created a ‘master list’ that retained the priority outcomes identified by each group. We cross-referenced these with the outcome survey already being utilized by the program to identify new ideas/priorities for outcome measurement and presented it to the leadership team.

See Table 3 for a comparison of project characteristics across projects 1 and 2.

**Table 3:** Comparing methods across project 1 and 2

Characteristic	Project 1: Day Health Program	Project 2: Safer Opioid Supply Program
Aim	Build capacity and collaboratively develop community-relevant health outcome and process indicators for a forthcoming day health program at a specialized care hospital.	Build capacity of stakeholders to recommend health outcome indicators to supplement the program’s existing six-monthly outcome survey.
Setting & Population	Specialized care hospital (13 inpatient beds; ~100 outpatient PLHIV-WUD).	Community-based safer opioid supply program (~ 150 clients at risk of overdose).
Stakeholder Groups	Clients (current/former inpatients), staff/clinicians, managers/senior leadership.	Clients, internal service providers (nurses, counsellors), external agency providers (social workers).
Recruitment	Flyers/posters in hospital; staff distribution; hospital listserv for staff/management.	Flyers in program; referrals via leadership; support for clients without email.
Sample Size	• 18 clients (14 attended ≥ 2 sessions; 9 attended all 4; 52 total client attendances across all sessions), • 10 staff, 3 managers • 31 total participants	• 8 clients, • 7 internal providers, • 8 external providers • 23 total participants.
Session Format & Count	• Clients: four sequential in-person sessions (1.5–2 h each); • Staff/Managers: one condensed session each (≈ 2 h)	• Clients/Internal: in-person ; 1 ~ 1.5 h • External: Zoom + online whiteboard/ dotmocracy; 1–1.5 h
Facilitation	• Co-facilitated by research coordinator (KR) and a peer-research assistant.	• Co-facilitated by CS and AG.
Activities	• Didactic on indicators; • Brainstorming; • Dotmocracy prioritization;	• Didactic overview; • Brainstorming; • Dotmocracy (sticky dots/Jamboard); • Audio-recording;•
Data Collection & Analysis	• Audio recording • Pictures/screen shots of dotmocracy • Content analysis	• Audio recording • Pictures/screen shots of dotmocracy • Content analysis
Incentives	• Clients: $30 CAD + 2 transit tickets + snacks; • Staff/Managers: snacks only.	• Clients: $30 CAD + snacks; • Providers: snacks
Main Outputs	• Final master list of prioritized indicators delivered to team for system development.	• Five new outcome domains added to the client survey; • Recommendations integrated into the program’s monitoring system.
Timeframe	• Sessions: May–June 2017;	• All sessions: November 2021; prioritization entirely within these sessions (no delayed follow-up).

## Results

### Project 1 – day health program

For study 1, we recruited *n* = 18 clients with *n* = 14 attending 2 or more sessions and *n* = 9 attending all 4 unique sessions for a total of 52 attendees across all client sessions. We recruited *n* = 10 staff/clinicians and *n* = 3 senior leadership/managers for one session each. Throughout the sessions participants were keen to share their thoughts about outcome measurement. Clients especially appreciated the opportunity to discuss their desires and needs for the day health program as well as having a space to voice their concerns and discuss how to evaluate the success of the program for them.

During the session where participants were shown the mandated outcome indicators for the program, clients suggested and prioritized some additional indicators, including: quality of life inclusive of housing, food and financial security; mental health; satisfaction with pain management; access to harm reduction services; social connectedness; and ease of readmission for those with frequent episodic illnesses. When asked about preferred method for collecting outcome data, clients preferred face-to-face, peer administered qualitative interviews.

Key staff -identified outcome indicators included: social determinants of health (housing, poverty, nutrition); access/continuity of care and case management after discharge; access to mental health services; sense of connectedness and belonging to the hospital; trust in hospital/program staff; and assistance with referrals and external appointments. For senior leadership/managers, key indicators included: social determinants of health (housing stability, food security), mental health and trauma assessment; pain management approaches and effectiveness; quality and clinical impact of care; client experience beyond simple satisfaction metrics; and continuum of care coordination (e.g., service engagement, transitions, post-discharge care, and collaboration with other service organizations). Senior leadership/managers recognized the need for a balance between using standard tools (brief/short) and one-on-one conversations to assess key indicators. After all the lists from the consultation sessions were finalized, the research team provided the final ‘master list’ created by the client group to the senior leadership/management team to be given to an external consulting agency developing the outcome monitoring system.

### Project 2 - Safer opioid supply program

For study 2, 8 program clients, 7 internal program providers, and 8 external agency service providers participated in one session each. All participants were enthusiastic to share their thoughts about indicators important to evaluate safer opioid program success. A total of 5 new outcome domains were recommended by participants to be added to the client outcome survey. Suggested additional outcome domains to measure included experiences of social isolation and connectedness (i.e., reconnecting with family and friends), experiences of discrimination in the program and in other health and service setting, changes in client self-care activities, client hobbies and leisure activities, and client experiences of grief and loss. Clients and staff emphasized the need to measure social outcomes in addition to health outcomes. A unique reflection made by staff included the need to measure program-level outcomes in addition to client-level outcomes. Staff wanted to monitor and evaluate partnerships with community agencies.

Clients shared that they preferred semi-structured interviews or the use of existing electronic health record data for collection of outcome indicator data whereas staff preferred using focus groups or semi-structured interviews to collect additional outcome indicators.

## Conclusions

This paper offers insight into how collaborative outcome indicator sessions were designed and delivered with people who use drugs and were at risk of or living with HIV. Often, client priorities for measuring program success, particularly for people who use drugs, are not included in traditional outcome indicators or evaluation measures [55]. While earlier studies have focused on indicator development through retrospective analysis [33, 35], our work emphasizes collaborative outcome indicator development, engaging people who use drugs directly in the design process. This shift from researcher-led to community-driven approaches reflects a deeper commitment to power-sharing, relevance, and accountability. By involving participants from the outset, including in facilitation and decision-making, we move beyond traditional models to co-create evaluation tools that better reflect lived experience and community priorities [56]. The implementation of client-centered programs can create tailored, flexible, and wrap around support which align with trauma and violence informed ideologies and can help reduce harms faced by these groups [57].

These two projects provided an opportunity for us to develop and refine a research method geared towards supporting those who are often excluded from decision-making regarding how to measure program success (or failure). Our experiences demonstrate great potential for an interactive, participant driven approach to outcome indicator development. In all sessions, participants were actively engaged in the capacity building and brainstorming activities. There are several lessons we have gathered from both the projects presented in this paper.

The primary lesson is the importance of integrating capacity building activities into collaborative indicator development sessions. Our approach included capacity building components to help participants understand the concept and purpose of outcome indicators, how indicators are created, why they are essential for evaluating program effectiveness, and how data about indicators can be collected. The collaborative nature of our approach was evident in how participants worked together during sessions to generate ideas and identify priorities. Within each project, clients, staff, and leadership teams all contributed valuable input toward enhancing program performance measurements. This integration of capacity building established the foundation of knowledge and skills that enabled meaningful engagement, fostering both confidence to contribute and sustained participation throughout the process [58]. The approach created space for personal reflection among participants, which ultimately strengthened both the quality of the indicators developed and participants’ investment in their implementation. These educational elements equipped participants to recommend indicators that were not only meaningful to them but also practical to implement. The output of the sessions from project 1 facilitated the identification of a list of key indicators that were then given to the team finalizing the indicators for the day health program electronic medical record. Discussions also occasionally moved beyond indicator development, revealing participants’ strong emotional attachment to the hospital as a place of safety, stability, and belonging. Although not outcome indicators themselves, these insights offered valuable program-level understanding about how hospital-based services can meet broader relational and restorative needs of clients facing structural vulnerability [59]. The output of the condensed sessions in project 2 led to the generation of practical recommendations which staff implemented directly into their outcome survey. These recommendations supported the collection of indicators which allowed for insight into clients’ overall well-being, such as measuring self-care, social connectedness, and leisure activities, which were not originally considered by program funders. These two projects allowed internal staff to adapt existing outcome measures to account for outcomes relevant for clients and service providers and offered an opportunity for reflection on the current services offered to clients and how desired outcomes, that fell outside of commonly implemented program measures, can be better met.

Key lessons also emerged regarding the optimal structure of outcome indicator development sessions. In the first project, we conducted multiple sequential sessions to build client capacity around outcome indicators. While attendance remained consistently high, participant feedback and our observations suggested that condensing these sessions could be effective. Our experience with staff and leadership teams confirmed this, demonstrating that the entire curriculum could be successfully compressed into a single session without compromising quality. This condensed format offers significant advantages for individuals experiencing housing instability or other forms of structural vulnerability, who may be unable or unwilling to commit to multiple sessions. The compressed structure proved especially valuable during COVID-19, when meeting restrictions limited in-person gatherings and stakeholders faced additional barriers to participation in multiple sessions. Additionally, as demonstrated in project 2, a more compact design allows for a greater number of sessions to be held, thereby expanding participation across a broader range of stakeholders and increasing the diversity of input into the indicator development process.

The final lesson relates to the opportunities and challenges of online engagement in outcome indicator development. We learned that holding virtual sessions may create new participation pathways for stakeholders who have limited capacity for in-person attendance, particularly program leadership. Through digital technology, we successfully adapted our brainstorming approach to the online environment. These virtual sessions produced valuable outputs; however, this format may not adequately serve those who prefer face-to-face collaboration or individuals experiencing structural vulnerability who lack appropriate space or technological means for virtual participation. For these groups, comprehensive support—such as providing access to computers and private, comfortable spaces —may be necessary to ensure their meaningful inclusion in the virtual indicator development process.

These projects and the method we present here respond to broader calls for the meaningful engagement of people who use drugs in research, program development, and evaluation [12, 55]. Pauly and colleagues for example, highlight how existing patient-oriented research frameworks in Canada (i.e., the Canadian Institutes of Health Research (CIHR) Strategy for Patient-Oriented Research) have gaps in supporting and acknowledging the needs and experiences of people who use drugs [60]. These frameworks remain limited in centering clients’ voices and perspectives on research or evaluation [60]. To comprehensively support people who use drugs, research must draw on community based participatory and trauma-informed approaches that prioritize opportunities for “co-learning” – an aspect that collaborative outcome indicator sessions uphold [60, 61]. The importance of such collaborative approaches is demonstrated by Meyerson et al. (2024), who developed an instrument to measure structural indicators of community-research partnerships [25]. The researchers’ iterative development process involved ongoing feedback from an Advocacy Board including people with lived drug use experience over a 3-month period, exemplifying the kind of sustained engagement necessary for meaningful collaboration rather than tokenistic consultation [25]. While Meyerson et al.’s work focused on evaluating the structural dynamics of research relationships themselves, it reinforces the critical importance of embedding community voice throughout all stages of the research and evaluation process [25]. Their framework’s emphasis on power sharing, coalition engagement, and community capacity development provides valuable insights for our approach to developing outcome indicators for service delivery, demonstrating how collaborative methodologies can be applied across different domains to ensure meaningful community partnership. Both projects were applied research initiatives launched after program implementation, after which our team was invited to support the development of community-driven evaluation tools in ways that were inclusive, empowering, and contextually responsive. Each project was rooted in deep community engagement, with a strong emphasis on power-sharing and lived experience. In the first study, individuals with lived experience participated throughout, facilitating sessions and shaping outcome indicators without edits or oversight from staff or managers. The second project, conducted during the COVID-19 pandemic, faced significant constraints in reconnecting with participants facing diverse forms of material inequity, which limited opportunities for participants to review or comment on the final indicators. Nonetheless, the insights shared by community members were meaningfully integrated into the broader evaluation of the program.

Moving forward, it is crucial to further develop and refine methods for involving people who use drugs in the development of outcome indicators, ensuring that their voices remain central to the process. Our experiences demonstrate that integrating capacity building components creates the foundation for meaningful participation, while offering flexible formats—both compressed single sessions and multiple sequential meetings—can accommodate diverse participant needs and circumstances. The adaptability of this approach to both in-person and virtual environments further expands accessibility, though additional supports remain necessary for those experiencing structural vulnerability. Future research should focus on exploring strategies for scaling up this approach by identifying best practices for diverse communities and evaluating the long-term impact of these sessions on client experience, service delivery, and effectiveness of programming. This work has profound implications for community involvement, creating a more inclusive, responsive, and effective framework for addressing the complex needs of people who use drugs and advancing truly client-centered program evaluation.

## Data Availability

All data are anonymized and stored by the corresponding author. These data cannot be shared publicly.

## References

[1] Ametaj AA, Smith AM, Valentine SE. A stakeholder-engaged process for adapting an evidence-Based intervention for posttraumatic stress disorder for peer delivery. Adm Policy Mental Health Mental Health Serv Res. 2021;48:793–809. 10.1007/s10488-021-01129-3 PMC8364475 33813717

[2] Hamilton AB, et al. Engaging multilevel stakeholders in an implementation trial of evidence-based quality improvement in VA women’s health primary care. Translational Behav Med. 2017;7(3):478–85. 10.1007/s13142-017-0501-5 PMC5645285 28585163

[3] El-Bassel N, et al. Using community engagement to implement evidence-based practices for opioid use disorder: A data-driven paradigm & systems science approach. Drug Alcohol Depend. 2021;222(108675):1–9. 10.1016/j.drugalcdep.2021.108675 PMC8058324 33757707

[4] Manalili K, et al. Co-designing person-centred quality indicator implementation for primary care in alberta: A consensus study. Res Involv Engagem. 2022;8(1):59. 36348406 10.1186/s40900-022-00397-z PMC9641306

[5] Smith S, et al. Person-centred quality indicators for Australian aged care assessment services: A mixed methods study. Res Involv Engagem. 2024;10(1):88. 39143622 10.1186/s40900-024-00606-x PMC11323374

[6] Skovlund SE, et al. The participatory development of a National core set of person-centred diabetes outcome constructs for use in routine diabetes care across healthcare sectors. Res Involv Engagem. 2021;7(1):62. 34507618 10.1186/s40900-021-00309-7 PMC8434700

[7] de Wit M et al. Involving patient research partners has a significant impact on outcomes research: a responsive evaluation of the international OMERACT conferences. BMJ Open, 2013. 3. 10.1136/bmjopen-2012-002241 PMC3651970 23667160

[8] Abma T, et al. Social impact of participatory health research: collaborative non-linear processes of knowledge mobilization. Educational Action Res. 2017;25(4):489–505. 10.1080/09650792.2017.1329092 PMC6101044 30135617

[9] Greer AM, et al. Peer engagement barriers and enablers: insights from people who use drugs in British Columbia, Canada. Can J Public Health. 2019;110(2):227–35. 30610564 10.17269/s41997-018-0167-x PMC6964581

[10] Greenhalgh T, et al. Achieving research impact through co-creation in community-based health services: literature review and case study. Milbank Q. 2016;94(2):392–429. 27265562 10.1111/1468-0009.12197 PMC4911728

[11] Salazar Z, et al. Research led by people who use drugs: centering the expertise of lived experience. Subst Abuse Treat Prev. 2021;16(70):1–4. 10.1186/s13011-021-00406-6 PMC8454046 34544478

[12] Harris M, Luongo N. *Nothing about us, without us: negotiating the personal and professional as activists and academics who use drugs* international. J Drug Policy. 2021;98(103533):1–4. 10.1016/j.drugpo.2021.103533 34895660

[13] Brown G, et al. Achieving meaningful participation of people who use drugs and their peer organizations in a strategic research partnership. Harm Reduct J. 2019;16(37):1–10. 31182099 10.1186/s12954-019-0306-6 PMC6558880

[14] Greer AM, et al. In: Control BCD, editor. Peer engagement best practices: A guide for health authorities and other providers. Editor: Vancouver, BC; 2017.

[15] Israel BA, et al. Chap. 3: Critical issues in developing and following CBPR principles. In: Wallerstein N, et al. editors. Community-based participatory research for health: advancing social and health equity. Wiley Brand: San Francisco; 2018.

[16] Minkler M, Wallerstein N. Community-based participatory research for health: from process to outcomes. Wiley; 2011.

[17] Flicker S et al. Community-based research in AIDS-service organizations: what helps and what doesn’t? AIDS Care, 2008. 21(1). 10.1080/09540120802032650 19085225

[18] Strike C, et al. Opportunities, challenges and ethical issues associated with conducting community-based participatory research in a hospital setting. Res Ethics. 2016;12(3):149–57.

[19] Damon W, et al. Community-based participatory research in a heavily researched inner City neighbourhood: perspectives of people who use drugs on their experiences as peer researchers. Soc Sci Med. 2017;21(176):85–92. 10.1016/j.socscimed.2017.01.027 PMC5438752 28135693

[20] Souleymanov R et al. The ethics of community-based research with people who use drugs: results of a scoping review. BMC Med Ethics 2016. 17(25). 10.1186/s12910-016-0108-2 PMC4850694 27129927

[21] Sandoval G, Rongerude J. Telling a story that must be heard: participatory indicators as tools for community empowerment. J Community Pract. 2015;23(3–4):403–14.

[22] Kotter T, et al. Involving patients in quality indicator development: A systematic review. Patient Prefer Adherence. 2013;7:259–68. 23569365 10.2147/PPA.S39803 PMC3616132

[23] Boivin A et al. Involving patients in setting priorities for healthcare improvement: a cluster randomized trial. Implement Sci, 2014. 9(24). 10.1186/1748-5908-9-24 PMC3936906 24555508

[24] Ball S, Rejon R. Unlocking policy success: is codesign the key to achieving effective policy outcomes? Policy Des Pract. 2024;7(3):251–64.

[25] Meyerson BE, et al. SI-CBPAR: towards structural indicators of community-based participatory action research. Drug Alcohol Rev. 2023;43(5):1049–61. 37872867 10.1111/dar.13764

[26] Wolff AC, et al. Healthcare provider characteristics that influence the implementation of individual-level patient-centered outcome measure (PROM) and patient-reported experience measure (PREM) data across practice settings: a protocol for a mixed methods systematic review with a narrative synthesis. Syst Reviews. 2021;10(169):1–12. 10.1186/s13643-021-01725-2 PMC8188663 34108024

[27] (CIHI). C.I.f.H.I. Patient-centred measurement and reporting in canada: launching the discussion toward a future state. CIHI: Ottawa; 2017.

[28] Boyce MB, Browne JP. Does providing feedback on patient reported outcomes to healthcare professionals result in better outcomes for patients? A systematic review. Qual Life Res. 2013;22(9):2265–78. 23504544 10.1007/s11136-013-0390-0

[29] Santana MJ, Feeny D. Framework to assess the effects of using patient-reported outcome measures in chronic care management. Qual Life Res. 2014;23(5):1505–13. 24318085 10.1007/s11136-013-0596-1

[30] Madden A, et al. Acceptability of Patient-reported outome and experience measures for hepatitis C treatment among people who use drugs. Volume 12. The patient- patient- patient-centered outcomes research; 2019. pp. 259–65. 10.1007/s40271-018-0332-6 30270403

[31] Madden A, et al. Patient-reported measures as a justice project through involvement of service-user researchers. Practical justice: principles, practice and social change. Routledge; 2019.

[32] Olding M, et al. *Developing a patient-reported experience questionnaire with and for people who use drugs: A community engagement process in vancouver’s downtown Eastside* international. J Drug Policy. 2018;59:16–23. 10.1016/j.drugpo.2018.06.003 29966804

[33] Migchels C, et al. Patient reported outcome and experience measures (PROMs and PREMs) in substance use disorder treatment services: A scoping review. Drug Alcohol Depend. 2023;253(111017):1–10. 10.1016/j.drugalcdep.2023.111017 37995391

[34] van der Sterren A, et al. Involvement of people who use alcohol and other drug services in the development of patient-reported measures of experience: A scoping review. Health Expect. 2023;26(6):2109–709. 37515528 10.1111/hex.13829 PMC10632652

[35] Wiessing L, et al. *Monitoring quality and coverage of harm reduction services for people who use drugs: a consensus study* harm. Reduct J. 2018;14(19):1–14. 10.1186/s12954-017-0141-6 PMC5401609 28431584

[36] Rogers SJ, Ruefli T. Does harm reduction programming make a difference in the lives of highly marginalized, at-risk drug users? Harm Reduct J, 2004. 1(7). 10.1186/1477-7517-1-7 PMC420490 15171790

[37] Minkler M. Community-based research partnerships: challenges and opportunities. J Urb Health. 2005;82:ii3–12. 10.1093/jurban/jti034 PMC3456439 15888635

[38] Wood L. Participatory action learning and action research. London: Routledge; 2019.

[39] Suter E, et al. Indicators and measurement tools for health systems integration: A knowledge synthesis. Int J Integr Care. 2017;17(4):1–14. 10.5334/ijic.3931 PMC5854167 29588637

[40] Makhoul J, Nakkash R. Understanding youth: using qualitative methods to verify quantitative community indicators. Health Promot Pract. 2009;10(1):128–35. 17971480 10.1177/1524839907301423

[41] de Wit M, et al. Successful Stepwise development of patient research partnership: 14 years’ experience of actions and consequences in outcome measures in rheumatology (OMERACT). Patient. 2017;10:141–52. 27704486 10.1007/s40271-016-0198-4 PMC5362656

[42] Napoles AM, Santoyo-Olsson J, Stewart AL. Methods for translating evidence-based behavioral interventions for health-disparity communities. Prev Chronic Disease. 2013;21(10):1–10. 10.5888/pcd10.130133 PMC3839588 24262025

[43] Asare-Kyel D, Kloos J, Renaud FG. Multi-scale participatory indicator development approaches for climate change risk assessment in West Africa. Int J Disaster Risk Reduct. 2015;11:13–34.

[44] Mascarenhas A, Nunes LM, Ramos TB. Selection of sustainability indicators for planning: combining stakeholders’ participation and data reduction techniques. J Clean Prod. 2015;92:295–307.

[45] Donatuto J, Campbell L, Gregory R. Developing responsive indicators of indigenous community health. Int J Environ Res Public Health, 2016. 10.3390/ijerph13090899 PMC5036732 27618086

[46] Health Council of Canada. A citizen’s guide to health indicators: A reference guide for Canadians 2011: Toronto p. 44.

[47] Graham S. Tools for action series: A resource guide for designing a community indicator project SPARC BC., editor. Vancouver; 2008. p. 84.

[48] Penchelon D. The good indicators guide: Understanding how to use and choose indicators N.I.f.I.a. Improvement, editor. United Kingdom; 2017. p. 40.

[49] First Nations Centre., Understanding health indicators., N.A.H. Organization, Editor. 2007: Ottawa. p. 26.

[50] Diceman J. Dotmocracy handbook: A simple tool to help large groups find agreement. Saatavilla. 2010;22(p):2013.

[51] Rudzinski K, et al. And if my goal is never to leave casey house? The significance of place attachment for patients at a specialty HIV hospital in Toronto, Canada. Volume 83. Health & Place; 2023. pp. 1–8. 103100. 10.1016/j.healthplace.2023.103100 37595542

[52] Hsieh HF, Shannon SE. Three approaches to qualitative content analysis. Qual Health Res. 2005;15(9):1277–88. 16204405 10.1177/1049732305276687

[53] Ivsins A, et al. Tackling the overdose crisis: the role of safe supply. Int J Drug Policy. 2020;80:102769. 10.1016/j.drugpo.2020.102769. 32446183 10.1016/j.drugpo.2020.102769 PMC7252037

[54] Perri M, et al. Outcomes from the safer supply program in Kitchener-Waterloo: report 1. Sanguen: Kitchener Waterloo; 2023.

[55] Perri M, et al. A rapid review of current engagement strategies with people who use drugs in monitoring and reporting on substance use-related harms. Harm Reduct J. 2023;20(1):169. 37964286 10.1186/s12954-023-00902-x PMC10648706

[56] Rudzinski K, et al. Twelve characteristics of client-centred supervised consumption services (SCS): a toolkit for service design, delivery and evaluation. Toronto; 2022.

[57] Robinson K, Ickowicz S. Research with women who use drugs: applying a trauma-informed framework. J Addict Med. 2022;16(6):627–9. 35678457 10.1097/ADM.0000000000000998 PMC9970160

[58] Kryszajtys DT et al. Do mock-ups, presentations of evidence, and Q&As help participants voice their opinions during focus groups and interviews about supervised injection services? Int J Qualitative Methods, 2021. 20.

[59] Rudzinski K, et al. And if my goal is never to leave casey house? The significance of place attachment for patients at a specialty HIV hospital in Toronto, Canada. Volume 83. Health & Place; 2023. pp. 1–10. 103100. 10.1016/j.healthplace.2023.103100 37595542

[60] Pauly B, et al. Applicability of a National strategy for patient-oriented research to people who use(d) substances: A Canadian experience. Res Involv Engagem. 2022;8(22):1–14. 35610726 10.1186/s40900-022-00351-z PMC9127478

[61] Stone T, et al. Trauma-Informed principles in practice: A Mixed-Method study of Co-Producing systems change with people who have experienced multiple disadvantage. Health Expect. 2025;28(6):e70472. 41178210 10.1111/hex.70472 PMC12580506

